# Supplementary material for: Widespread increase in dynamic imbalance in the Getz region of Antarctica from 1994 to 2018
Source: Nat Commun. 2021 Feb 23;12:1133. doi: 10.1038/s41467-021-21321-1 (PMC7902837; doi:10.1038/s41467-021-21321-1)
Supplement: Supplementary file 1 — Supplementary Infomation [file 41467_2021_21321_MOESM1_ESM.pdf]

Supplementary Information

Widespread increase in dynamic imbalance in the Getz region of Antarctica from 1994 to 2018

Selley et al.

***Supplementary Table 1**. Input satellite data used to measure ice speed from 1994 to 2018, for all 16 annual maps. The percent and area of the Getz drainage basin covered by satellite observations is also stated for each annual velocity map, along with the mean ice speed across the basin.*

Annual Velocity Map Name	Satellite	Data Type	Time Period Covered (YYYY.MM.DD)		Percentage Cover (%)	Area Covered (km²)	Mean Velocity (m/yr)
1994	ERS	SAR	1994.01.28	1994.02.03	4	7,316	193
1996			1996.01.26	1996.02.15	8	14,371	292
1998			1998.02.02	1998.06.13	2	3,432	221
2005	MEaSUREs data represent an average of the following satellite imagery: ALOS, ENVISAT, LANDSAT-8, RADARSAT-1, RADARSAT-2, SENTINEL-1A, SENTINEL-1B, TDX and TSX	SAR and Optical	2005.06.01	2006.05.30	41	71,305	218
2006			2006.06.01	2007.05.30	40	69,368	195
2007			2007.06.01	2008.05.30	65	111,373	168
2008			2008.06.01	2009.05.30	60	103,667	179
2009			2009.06.01	2010.05.30	28	48,183	202
2010			2010.06.01	2011.05.30	44	75,860	197
2012			2012.06.01	2013.05.30	11	18,458	283
2013			2013.06.01	2014.05.30	78	134,331	151
2014			2014.06.01	2015.05.30	90	154,209	130
2015			2015.06.01	2016.05.30	86	148,595	135
2016			2016.06.01	2017.05.30	90	155,518	132
2017	Sentinel-1	SAR	2017.02.07	2017.12.29	36	62,483	256
2018			2018.01.04	2018.12.26	60	102,466	201

Supplementary Fig. 1a. *A set of close up maps and velocity plots for flow unit 1 in the Getz study region. a) Profiles of ice speed from 1994 (dark blue) to 2018 (light blue) across a flux gate profile located at the grounding line (see white line in (c)) orientated perpendicular to the direction of ice flow. b) Bed topography (brown shaded area) and ice thickness (light blue shaded area) extracted along the same flux gate profile from BEDMAP2[2]. Annual ice surface elevation change from 1992 (black) to 2017 (yellow) is also shown using the five yearly rates of elevation change[3] using BEDMAP2 elevation as the reference year 2000. c) A close up of the 2018 observed ice velocity map for this glacier, with the flow unit (black dashed line) and the flux gate (thick white line) profile locations annotated. The grounding line location from 1992 (grey) to 2017 (pink) is also shown where measurements exist, and key topographic features such as ice rises (brown) are also shown. d) Change in ice speed (%) from 1994 to 2018 (black dots), calculated as the difference in speed from the 2018 measurement, using the mean speed from a 5 km diameter region where the flow unit crosses the grounding line. Data points that exceed the standard error are denoted by a black hollow dot and excluded from further trend analysis. The surface elevation change measured is relative to the WGS84 ellipsoid from radar altimetry data[3] using a 25 km diameter region at the intersection of the flow unit and grounding line also shown (blue triangles).*

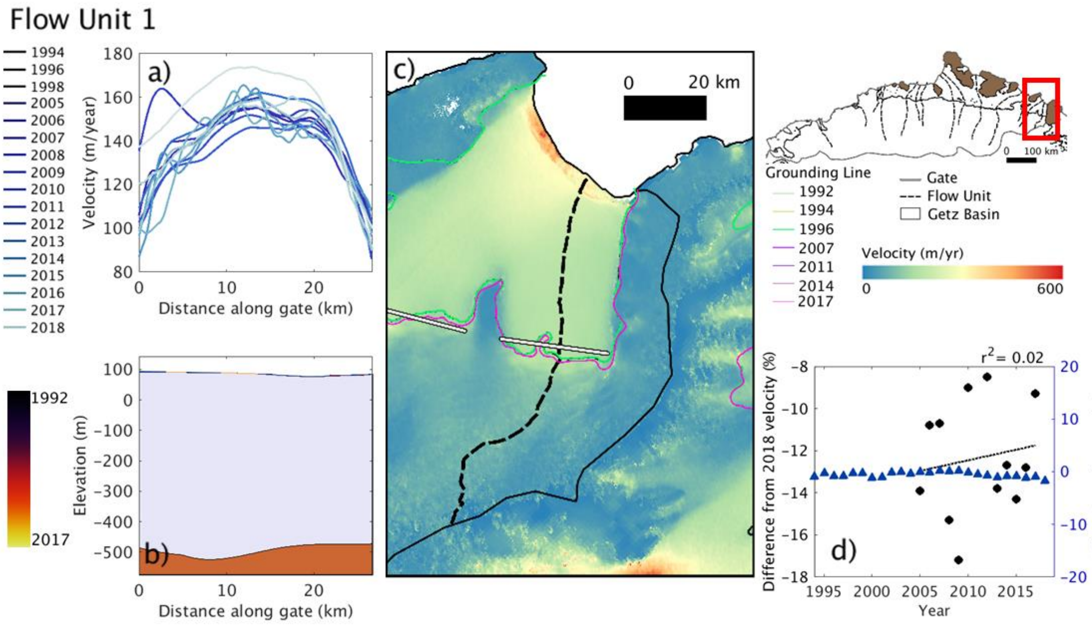

Supplementary Fig. 1b. *A set of close up maps and velocity plots for flow unit 2 in the Getz study region. a) Profiles of ice speed from 1994 (dark blue) to 2018 (light blue) across a flux gate profile located at the grounding line (see white line in (c)) orientated perpendicular to the direction of ice flow. b) Bed topography (brown shaded area) and ice thickness (light blue shaded area) extracted along the same flux gate profile from BEDMAP2[2]. Annual ice surface elevation change from 1992 (black) to 2017 (yellow) is also shown using the five yearly rates of elevation change[3] using BEDMAP2 elevation as the reference year 2000. c) A close up of the 2018 observed ice velocity map for this glacier, with the flow unit (black dashed line) and the flux gate (thick white line) profile locations annotated. The grounding line location from 1992 (grey) to 2017 (pink) is also shown where measurements exist, and key topographic features such as ice rises (brown) are also shown. d) Change in ice speed (%) from 1994 to 2018 (black dots), calculated as the difference in speed from the 2018 measurement, using the mean speed from a 5 km diameter region where the flow unit crosses the grounding line. Data points that exceed the standard error are denoted by a black hollow dot and excluded from further trend analysis. The surface elevation change measured is relative to the WGS84 ellipsoid from radar altimetry data[3] using a 25 km diameter region at the intersection of the flow unit and grounding line also shown (blue triangles).*

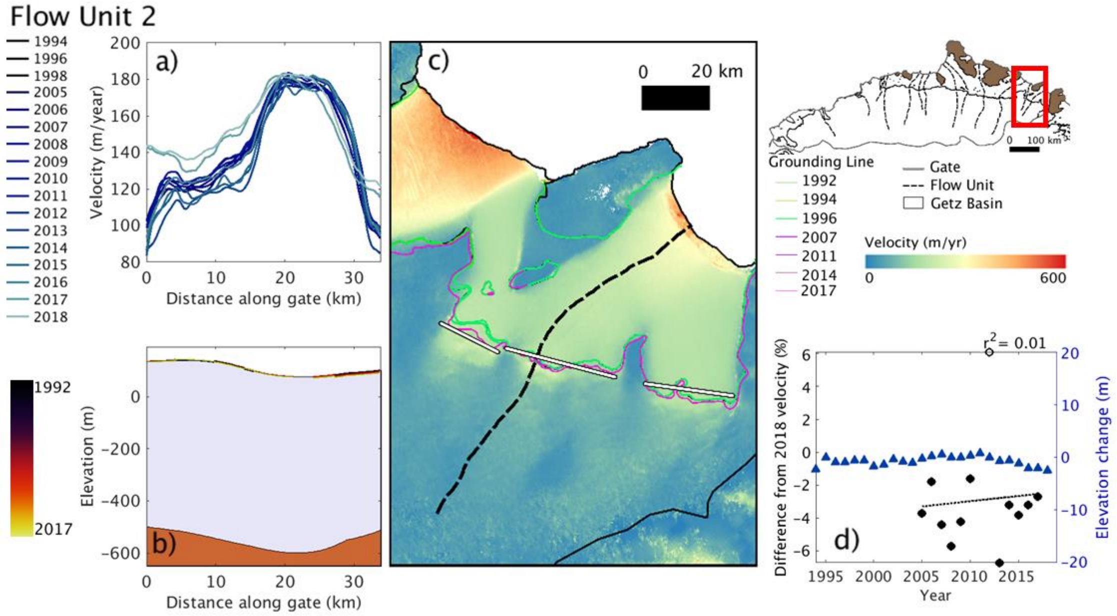

Supplementary Fig. 1c. *A set of close up maps and velocity plots for flow unit 3 in the Getz study region. a) Profiles of ice speed from 1994 (dark blue) to 2018 (light blue) across a flux gate profile located at the grounding line (see white line in (c)) orientated perpendicular to the direction of ice flow. b) Bed topography (brown shaded area) and ice thickness (light blue shaded area) extracted along the same flux gate profile from BEDMAP2[2]. Annual ice surface elevation change from 1992 (black) to 2017 (yellow) is also shown using the five yearly rates of elevation change[3] using BEDMAP2 elevation as the reference year 2000. c) A close up of the 2018 observed ice velocity map for this glacier, with the flow unit (black dashed line) and the flux gate (thick white line) profile locations annotated. The grounding line location from 1992 (grey) to 2017 (pink) is also shown where measurements exist, and key topographic features such as ice rises (brown) are also shown. d) Change in ice speed (%) from 1994 to 2018 (black dots), calculated as the difference in speed from the 2018 measurement, using the mean speed from a 5 km diameter region where the flow unit crosses the grounding line. Data points that exceed the standard error are denoted by a black hollow dot and excluded from further trend analysis. The surface elevation change measured is relative to the WGS84 ellipsoid from radar altimetry data[3] using a 25 km diameter region at the intersection of the flow unit and grounding line also shown (blue triangles).*

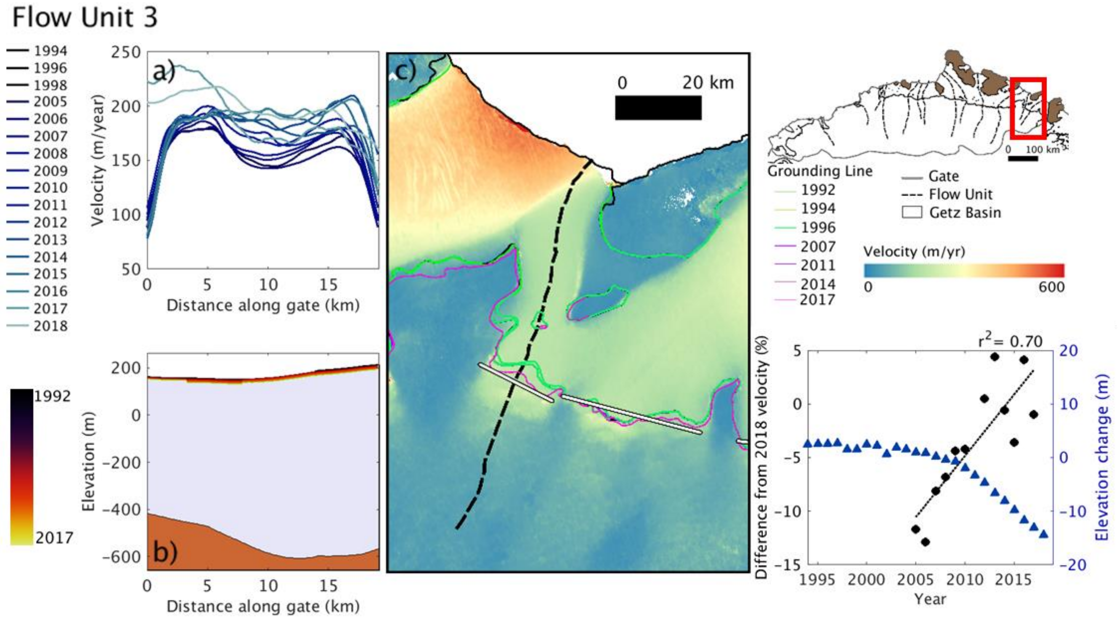

Supplementary Fig. 1d. *A set of close up maps and velocity plots for flow unit 4 in the Getz study region. a) Profiles of ice speed from 1994 (dark blue) to 2018 (light blue) across a flux gate profile located at the grounding line (see white line in (c)) orientated perpendicular to the direction of ice flow. b) Bed topography (brown shaded area) and ice thickness (light blue shaded area) extracted along the same flux gate profile from BEDMAP22. Annual ice surface elevation change from 1992 (black) to 2017 (yellow) is also shown using the five yearly rates of elevation change3 using BEDMAP2 elevation as the reference year 2000. c) A close up of the 2018 observed ice velocity map for this glacier, with the flow unit (black dashed line) and the flux gate (thick white line) profile locations annotated. The grounding line location from 1992 (grey) to 2017 (pink) is also shown where measurements exist, and key topographic features such as ice rises (brown) are also shown. d) Change in ice speed (%) from 1994 to 2018 (black dots), calculated as the difference in speed from the 2018 measurement, using the mean speed from a 5 km diameter region where the flow unit crosses the grounding line. Data points that exceed the standard error are denoted by a black hollow dot and excluded from further trend analysis. The surface elevation change measured is relative to the WGS84 ellipsoid from radar altimetry data3 using a 25 km diameter region at the intersection of the flow unit and grounding line also shown (blue triangles).*

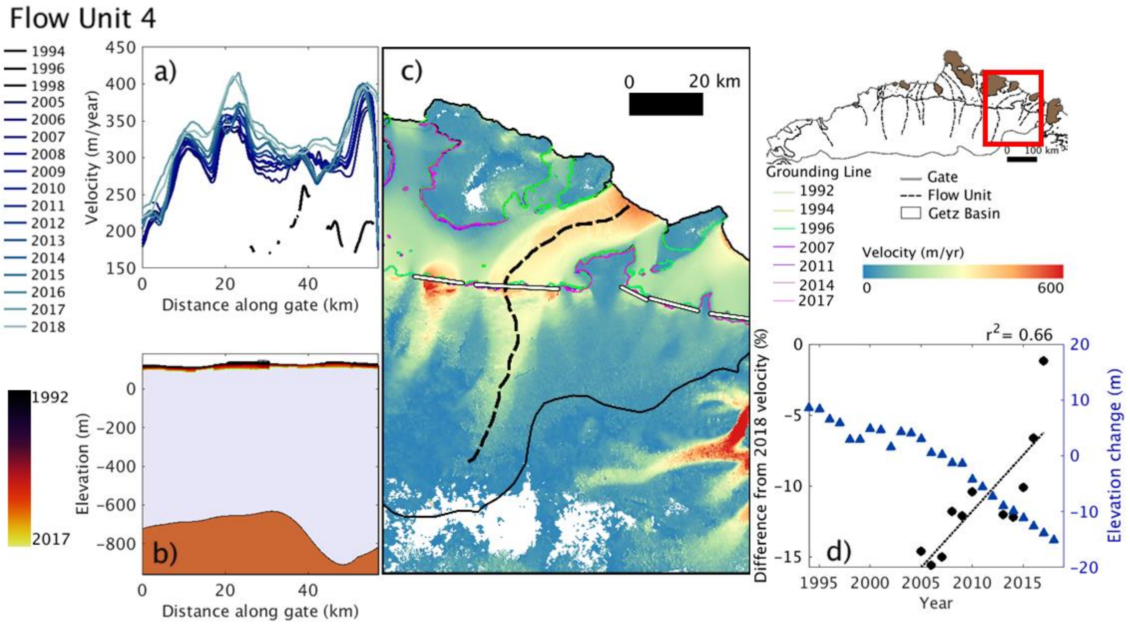

Supplementary Fig. 1e. *A set of close up maps and velocity plots for flow unit 5 in the Getz study region. a) Profiles of ice speed from 1994 (dark blue) to 2018 (light blue) across a flux gate profile located at the grounding line (see white line in (c)) orientated perpendicular to the direction of ice flow. b) Bed topography (brown shaded area) and ice thickness (light blue shaded area) extracted along the same flux gate profile from BEDMAP2[2]. Annual ice surface elevation change from 1992 (black) to 2017 (yellow) is also shown using the five yearly rates of elevation change[3] using BEDMAP2 elevation as the reference year 2000. c) A close up of the 2018 observed ice velocity map for this glacier, with the flow unit (black dashed line) and the flux gate (thick white line) profile locations annotated. The grounding line location from 1992 (grey) to 2017 (pink) is also shown where measurements exist, and key topographic features such as ice rises (brown) are also shown. d) Change in ice speed (%) from 1994 to 2018 (black dots), calculated as the difference in speed from the 2018 measurement, using the mean speed from a 5 km diameter region where the flow unit crosses the grounding line. Data points that exceed the standard error are denoted by a black hollow dot and excluded from further trend analysis. The surface elevation change measured is relative to the WGS84 ellipsoid from radar altimetry data[3] using a 25 km diameter region at the intersection of the flow unit and grounding line also shown (blue triangles).*

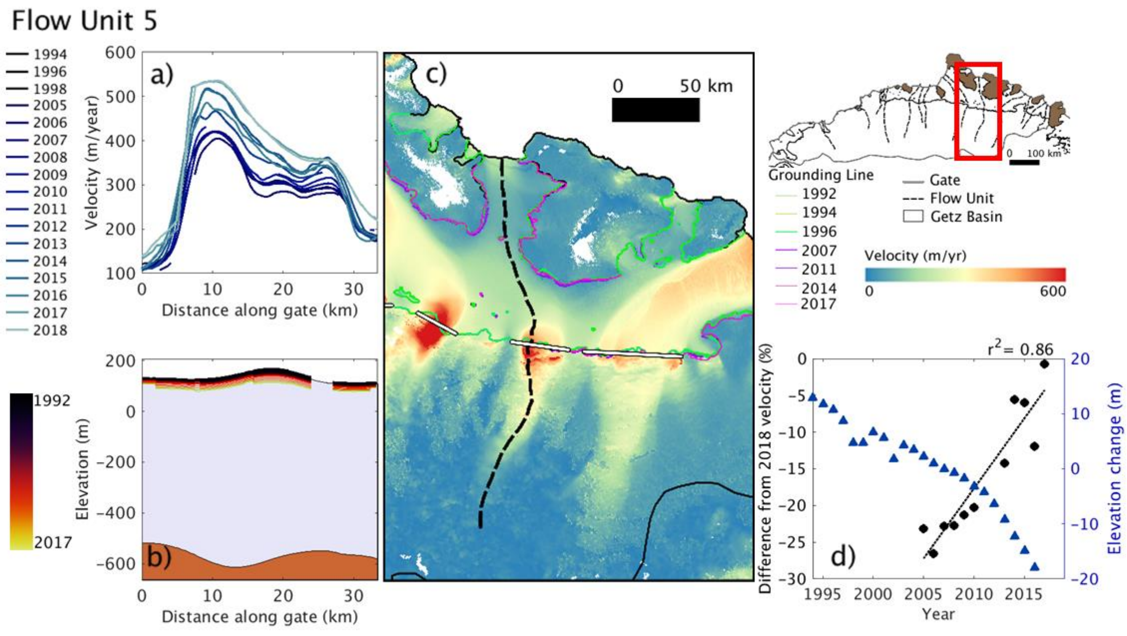

Supplementary Fig. 1f. *A set of close up maps and velocity plots for flow unit 6 in the Getz study region. a) Profiles of ice speed from 1994 (dark blue) to 2018 (light blue) across a flux gate profile located at the grounding line (see white line in (c)) orientated perpendicular to the direction of ice flow. b) Bed topography (brown shaded area) and ice thickness (light blue shaded area) extracted along the same flux gate profile from BEDMAP2[2]. Annual ice surface elevation change from 1992 (black) to 2017 (yellow) is also shown using the five yearly rates of elevation change[3] using BEDMAP2 elevation as the reference year 2000. c) A close up of the 2018 observed ice velocity map for this glacier, with the flow unit (black dashed line) and the flux gate (thick white line) profile locations annotated. The grounding line location from 1992 (grey) to 2017 (pink) is also shown where measurements exist, and key topographic features such as ice rises (brown) are also shown. d) Change in ice speed (%) from 1994 to 2018 (black dots), calculated as the difference in speed from the 2018 measurement, using the mean speed from a 5 km diameter region where the flow unit crosses the grounding line. Data points that exceed the standard error are denoted by a black hollow dot and excluded from further trend analysis. The surface elevation change measured is relative to the WGS84 ellipsoid from radar altimetry data[3] using a 25 km diameter region at the intersection of the flow unit and grounding line also shown (blue triangles).*

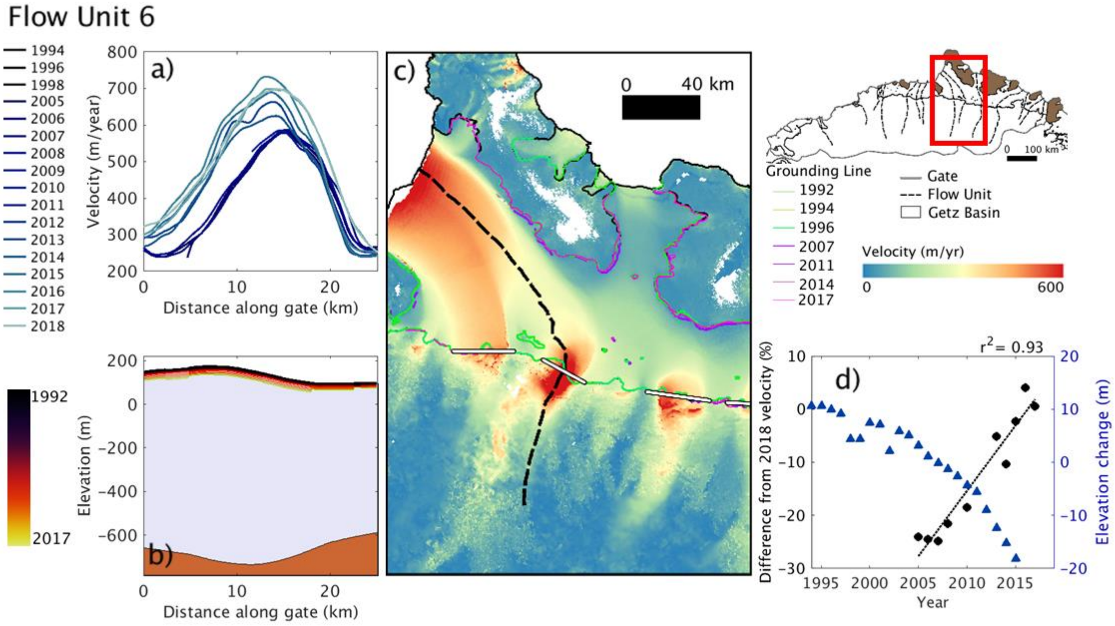

Supplementary Fig. 1g. *A set of close up maps and velocity plots for flow unit 7 in the Getz study region. a) Profiles of ice speed from 1994 (dark blue) to 2018 (light blue) across a flux gate profile located at the grounding line (see white line in (c)) orientated perpendicular to the direction of ice flow. b) Bed topography (brown shaded area) and ice thickness (light blue shaded area) extracted along the same flux gate profile from BEDMAP2[2]. Annual ice surface elevation change from 1992 (black) to 2017 (yellow) is also shown using the five yearly rates of elevation change[3] using BEDMAP2 elevation as the reference year 2000. c) A close up of the 2018 observed ice velocity map for this glacier, with the flow unit (black dashed line) and the flux gate (thick white line) profile locations annotated. The grounding line location from 1992 (grey) to 2017 (pink) is also shown where measurements exist, and key topographic features such as ice rises (brown) are also shown. d) Change in ice speed (%) from 1994 to 2018 (black dots), calculated as the difference in speed from the 2018 measurement, using the mean speed from a 5 km diameter region where the flow unit crosses the grounding line. Data points that exceed the standard error are denoted by a black hollow dot and excluded from further trend analysis. The surface elevation change measured is relative to the WGS84 ellipsoid from radar altimetry data[3] using a 25 km diameter region at the intersection of the flow unit and grounding line also shown (blue triangles).*

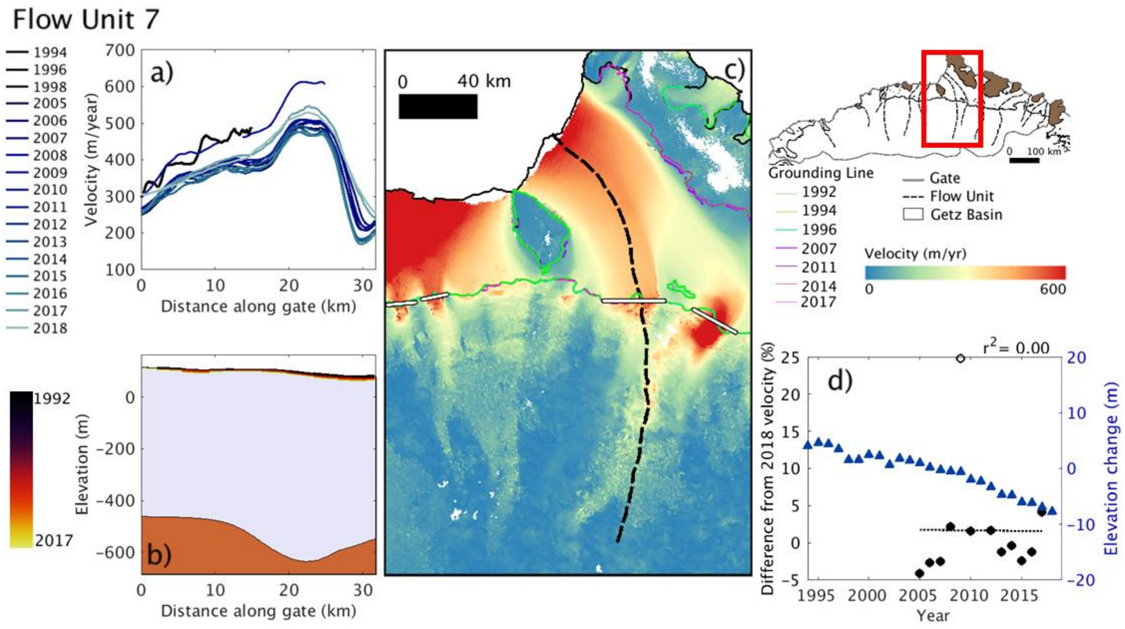

Supplementary Fig. 1h. *A set of close up maps and velocity plots for flow unit 8 in the Getz study region. a) Profiles of ice speed from 1994 (dark blue) to 2018 (light blue) across a flux gate profile located at the grounding line (see white line in (c)) orientated perpendicular to the direction of ice flow. b) Bed topography (brown shaded area) and ice thickness (light blue shaded area) extracted along the same flux gate profile from BEDMAP2[2]. Annual ice surface elevation change from 1992 (black) to 2017 (yellow) is also shown using the five yearly rates of elevation change[3] using BEDMAP2 elevation as the reference year 2000. c) A close up of the 2018 observed ice velocity map for this glacier, with the flow unit (black dashed line) and the flux gate (thick white line) profile locations annotated. The grounding line location from 1992 (grey) to 2017 (pink) is also shown where measurements exist, and key topographic features such as ice rises (brown) are also shown. d) Change in ice speed (%) from 1994 to 2018 (black dots), calculated as the difference in speed from the 2018 measurement, using the mean speed from a 5 km diameter region where the flow unit crosses the grounding line. Data points that exceed the standard error are denoted by a black hollow dot and excluded from further trend analysis. The surface elevation change measured is relative to the WGS84 ellipsoid from radar altimetry data[3] using a 25 km diameter region at the intersection of the flow unit and grounding line also shown (blue triangles).*

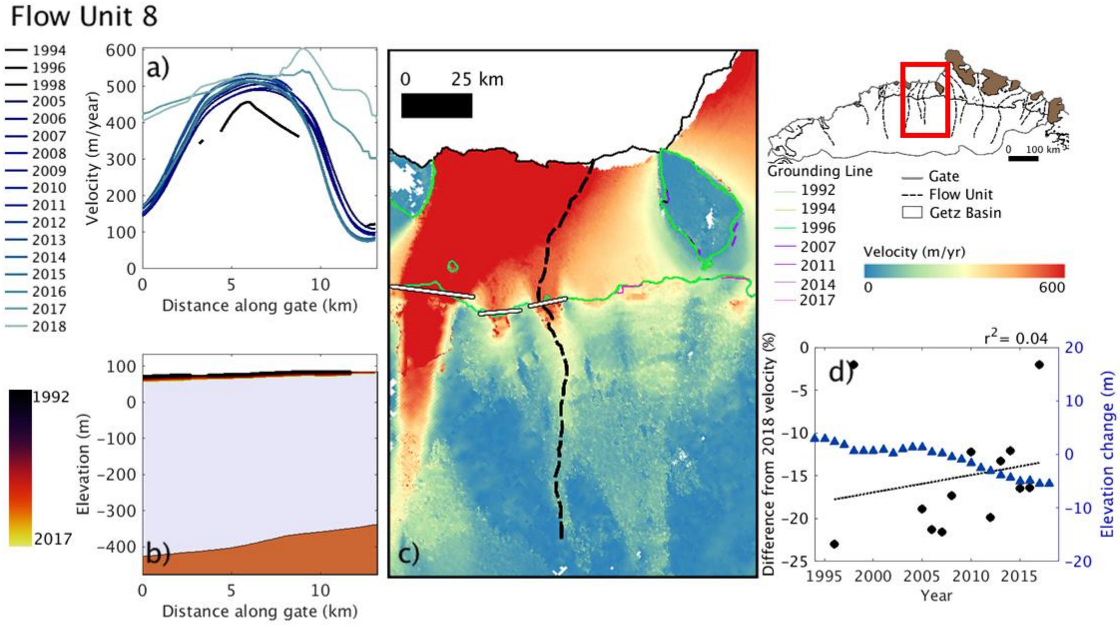

Supplementary Fig. 1i. *A set of close up maps and velocity plots for flow unit 9 in the Getz study region. a) Profiles of ice speed from 1994 (dark blue) to 2018 (light blue) across a flux gate profile located at the grounding line (see white line in (c)) orientated perpendicular to the direction of ice flow. b) Bed topography (brown shaded area) and ice thickness (light blue shaded area) extracted along the same flux gate profile from BEDMAP2[2]. Annual ice surface elevation change from 1992 (black) to 2017 (yellow) is also shown using the five yearly rates of elevation change[3] using BEDMAP2 elevation as the reference year 2000. c) A close up of the 2018 observed ice velocity map for this glacier, with the flow unit (black dashed line) and the flux gate (thick white line) profile locations annotated. The grounding line location from 1992 (grey) to 2017 (pink) is also shown where measurements exist, and key topographic features such as ice rises (brown) are also shown. d) Change in ice speed (%) from 1994 to 2018 (black dots), calculated as the difference in speed from the 2018 measurement, using the mean speed from a 5 km diameter region where the flow unit crosses the grounding line. Data points that exceed the standard error are denoted by a black hollow dot and excluded from further trend analysis. The surface elevation change measured is relative to the WGS84 ellipsoid from radar altimetry data[3] using a 25 km diameter region at the intersection of the flow unit and grounding line also shown (blue triangles).*

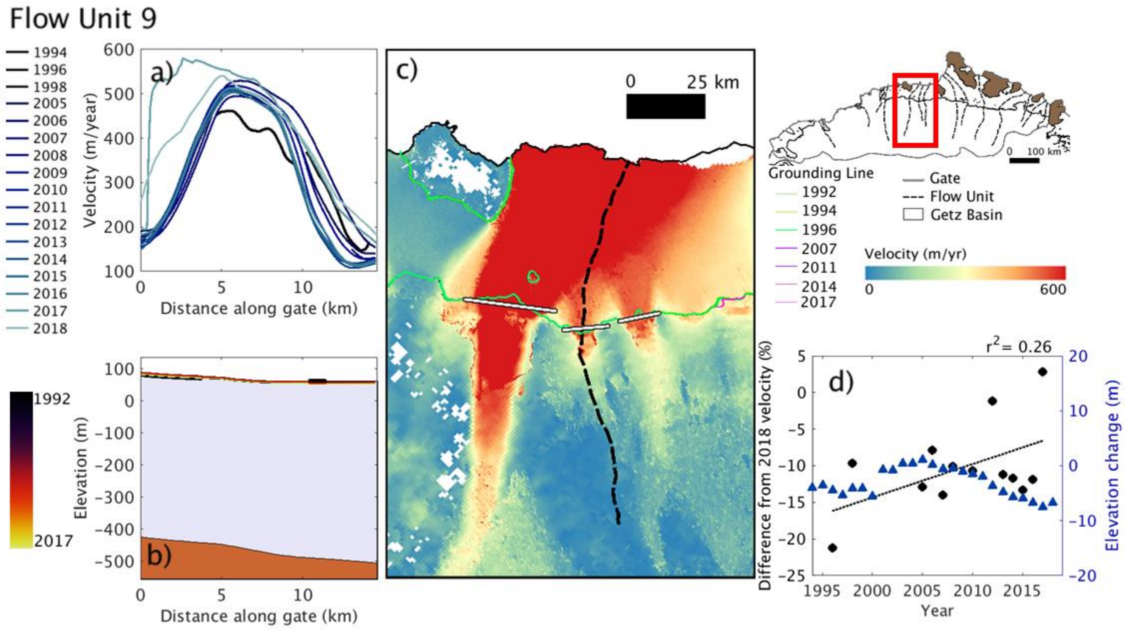

Supplementary Fig. 1j. *A set of close up maps and velocity plots for flow unit 10 in the Getz study region. a) Profiles of ice speed from 1994 (dark blue) to 2018 (light blue) across a flux gate profile located at the grounding line (see white line in (c)) orientated perpendicular to the direction of ice flow. b) Bed topography (brown shaded area) and ice thickness (light blue shaded area) extracted along the same flux gate profile from BEDMAP2[2]. Annual ice surface elevation change from 1992 (black) to 2017 (yellow) is also shown using the five yearly rates of elevation change[3] using BEDMAP2 elevation as the reference year 2000. c) A close up of the 2018 observed ice velocity map for this glacier, with the flow unit (black dashed line) and the flux gate (thick white line) profile locations annotated. The grounding line location from 1992 (grey) to 2017 (pink) is also shown where measurements exist, and key topographic features such as ice rises (brown) are also shown. d) Change in ice speed (%) from 1994 to 2018 (black dots), calculated as the difference in speed from the 2018 measurement, using the mean speed from a 5 km diameter region where the flow unit crosses the grounding line. Data points that exceed the standard error are denoted by a black hollow dot and excluded from further trend analysis. The surface elevation change measured is relative to the WGS84 ellipsoid from radar altimetry data[3] using a 25 km diameter region at the intersection of the flow unit and grounding line also shown (blue triangles).*

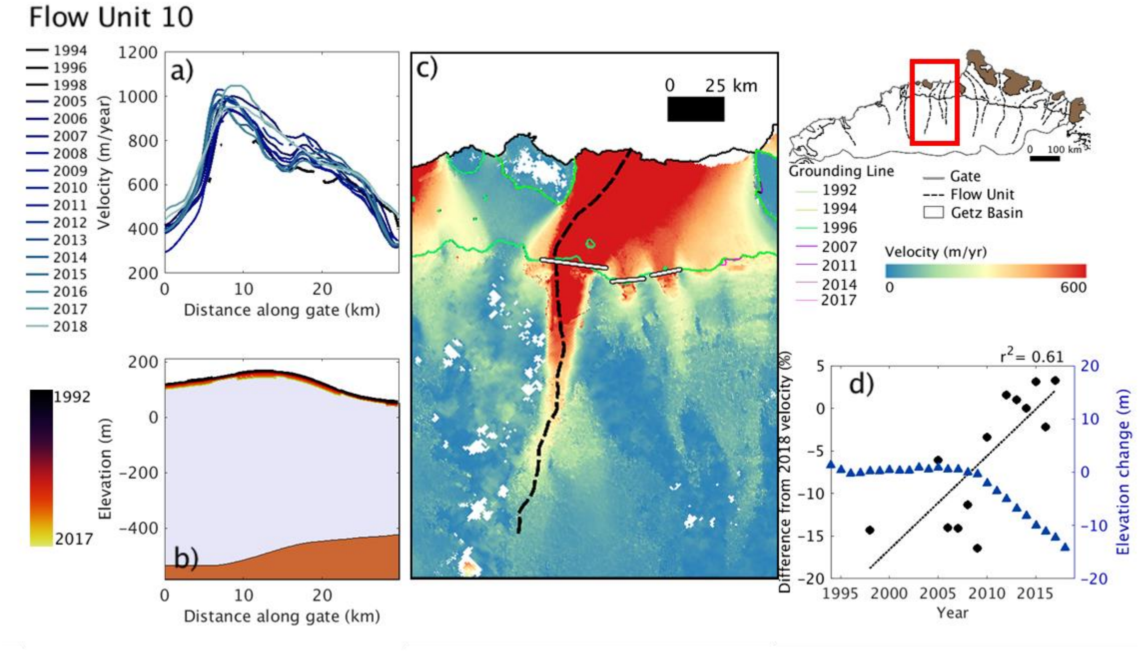

Supplementary Fig. 1k. *A set of close up maps and velocity plots for flow unit 11 in the Getz study region. a) Profiles of ice speed from 1994 (dark blue) to 2018 (light blue) across a flux gate profile located at the grounding line (see white line in (c)) orientated perpendicular to the direction of ice flow. b) Bed topography (brown shaded area) and ice thickness (light blue shaded area) extracted along the same flux gate profile from BEDMAP2[2]. Annual ice surface elevation change from 1992 (black) to 2017 (yellow) is also shown using the five yearly rates of elevation change[3] using BEDMAP2 elevation as the reference year 2000. c) A close up of the 2018 observed ice velocity map for this glacier, with the flow unit (black dashed line) and the flux gate (thick white line) profile locations annotated. The grounding line location from 1992 (grey) to 2017 (pink) is also shown where measurements exist, and key topographic features such as ice rises (brown) are also shown. d) Change in ice speed (%) from 1994 to 2018 (black dots), calculated as the difference in speed from the 2018 measurement, using the mean speed from a 5 km diameter region where the flow unit crosses the grounding line. Data points that exceed the standard error are denoted by a black hollow dot and excluded from further trend analysis. The surface elevation change measured is relative to the WGS84 ellipsoid from radar altimetry data[3] using a 25 km diameter region at the intersection of the flow unit and grounding line also shown (blue triangles).*

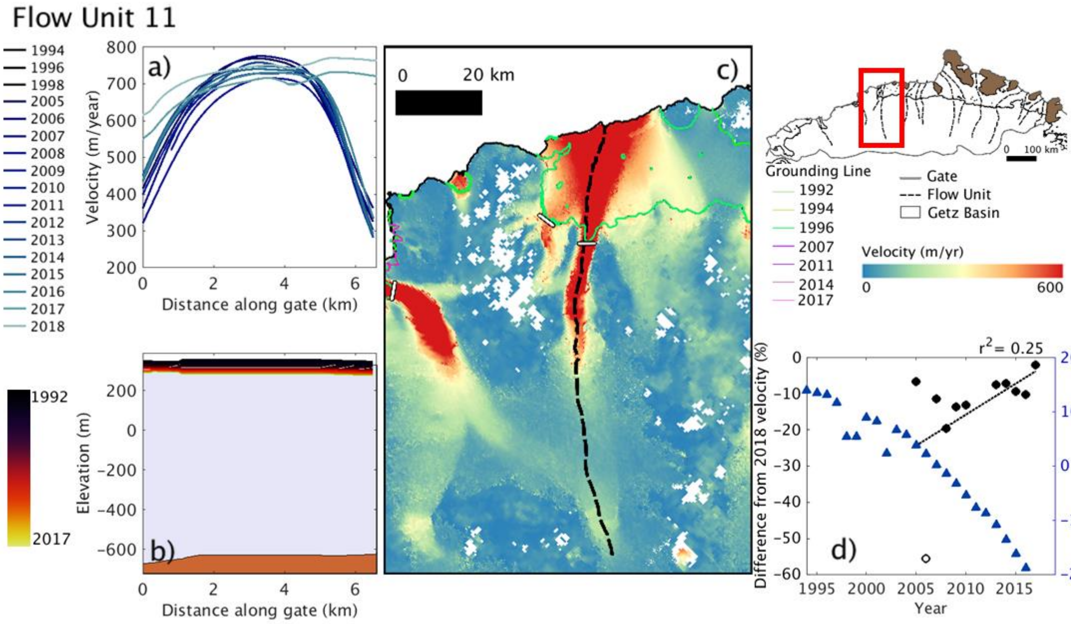

Supplementary Fig. 1l. *A set of close up maps and velocity plots for flow unit 12 in the Getz study region. a) Profiles of ice speed from 1994 (dark blue) to 2018 (light blue) across a flux gate profile located at the grounding line (see white line in (c)) orientated perpendicular to the direction of ice flow. b) Bed topography (brown shaded area) and ice thickness (light blue shaded area) extracted along the same flux gate profile from BEDMAP2[2]. Annual ice surface elevation change from 1992 (black) to 2017 (yellow) is also shown using the five yearly rates of elevation change[3] using BEDMAP2 elevation as the reference year 2000. c) A close up of the 2018 observed ice velocity map for this glacier, with the flow unit (black dashed line) and the flux gate (thick white line) profile locations annotated. The grounding line location from 1992 (grey) to 2017 (pink) is also shown where measurements exist, and key topographic features such as ice rises (brown) are also shown. d) Change in ice speed (%) from 1994 to 2018 (black dots), calculated as the difference in speed from the 2018 measurement, using the mean speed from a 5 km diameter region where the flow unit crosses the grounding line. Data points that exceed the standard error are denoted by a black hollow dot and excluded from further trend analysis. The surface elevation change measured is relative to the WGS84 ellipsoid from radar altimetry data[3] using a 25 km diameter region at the intersection of the flow unit and grounding line also shown (blue triangles).*

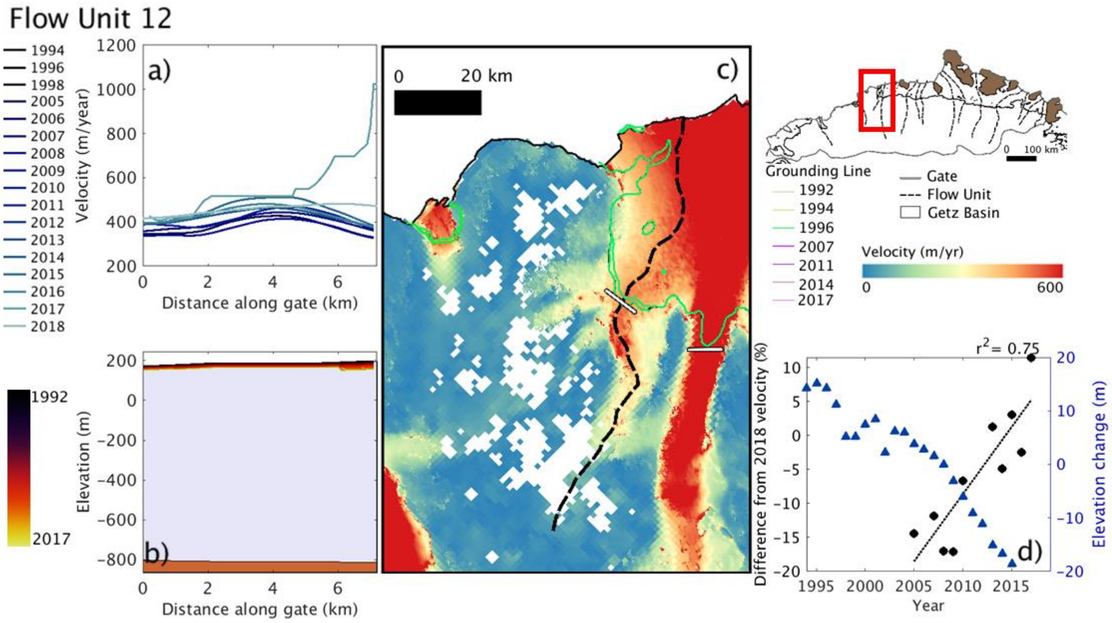

Supplementary Fig. 1m. *A set of close up maps and velocity plots for flow unit 13 in the Getz study region. a) Profiles of ice speed from 1994 (dark blue) to 2018 (light blue) across a flux gate profile located at the grounding line (see white line in (c)) orientated perpendicular to the direction of ice flow. b) Bed topography (brown shaded area) and ice thickness (light blue shaded area) extracted along the same flux gate profile from BEDMAP2[2]. Annual ice surface elevation change from 1992 (black) to 2017 (yellow) is also shown using the five yearly rates of elevation change[3] using BEDMAP2 elevation as the reference year 2000. c) A close up of the 2018 observed ice velocity map for this glacier, with the flow unit (black dashed line) and the flux gate (thick white line) profile locations annotated. The grounding line location from 1992 (grey) to 2017 (pink) is also shown where measurements exist, and key topographic features such as ice rises (brown) are also shown. d) Change in ice speed (%) from 1994 to 2018 (black dots), calculated as the difference in speed from the 2018 measurement, using the mean speed from a 5 km diameter region where the flow unit crosses the grounding line. Data points that exceed the standard error are denoted by a black hollow dot and excluded from further trend analysis. The surface elevation change measured is relative to the WGS84 ellipsoid from radar altimetry data[3] using a 25 km diameter region at the intersection of the flow unit and grounding line also shown (blue triangles).*

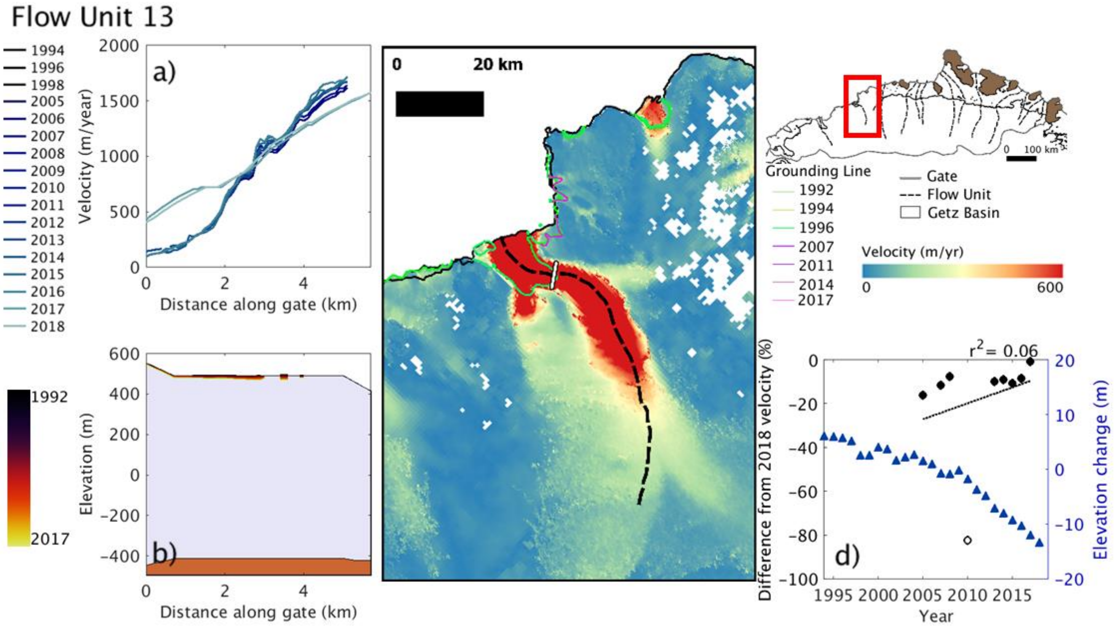

Supplementary Fig. 1n. *A set of close up maps and velocity plots for flow unit 14 in the Getz study region. a) Profiles of ice speed from 1994 (dark blue) to 2018 (light blue) across a flux gate profile located at the grounding line (see white line in (c)) orientated perpendicular to the direction of ice flow. b) Bed topography (brown shaded area) and ice thickness (light blue shaded area) extracted along the same flux gate profile from BEDMAP2[2]. Annual ice surface elevation change from 1992 (black) to 2017 (yellow) is also shown using the five yearly rates of elevation change[3] using BEDMAP2 elevation as the reference year 2000. c) A close up of the 2018 observed ice velocity map for this glacier, with the flow unit (black dashed line) and the flux gate (thick white line) profile locations annotated. The grounding line location from 1992 (grey) to 2017 (pink) is also shown where measurements exist, and key topographic features such as ice rises (brown) are also shown. d) Change in ice speed (%) from 1994 to 2018 (black dots), calculated as the difference in speed from the 2018 measurement, using the mean speed from a 5 km diameter region where the flow unit crosses the grounding line. Data points that exceed the standard error are denoted by a black hollow dot and excluded from further trend analysis. The surface elevation change measured is relative to the WGS84 ellipsoid from radar altimetry data[3] using a 25 km diameter region at the intersection of the flow unit and grounding line also shown (blue triangles).*

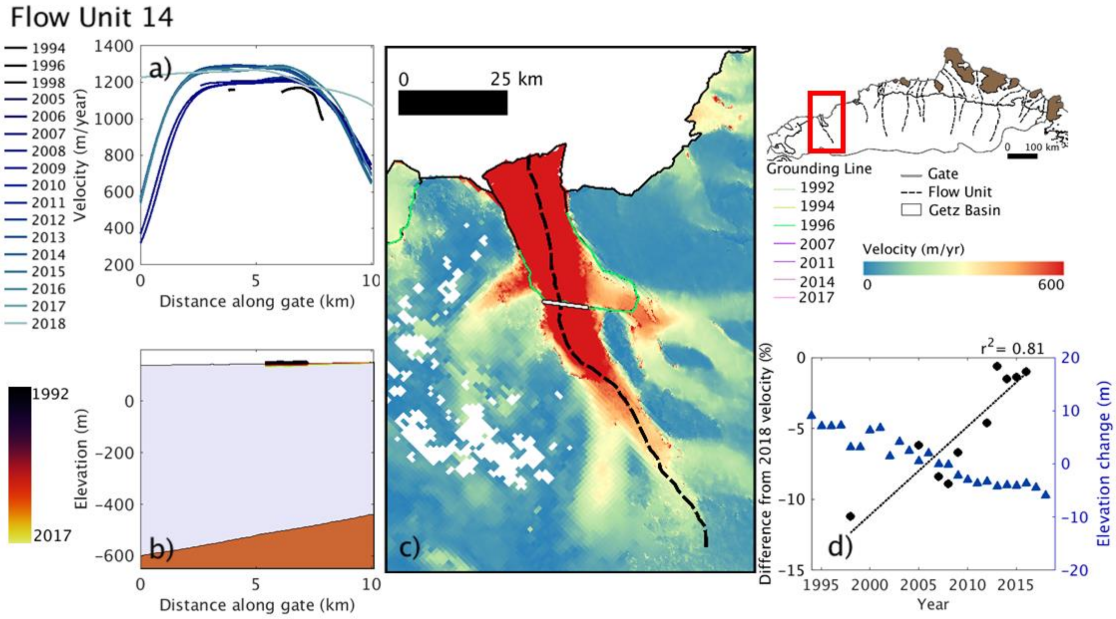

***Supplementary Fig. 2a**. The observed ice speed of the Getz drainage basin (left) and the associated error estimate (right), for 1994 to 1998 annual maps. The satellite observations included in each annual map are specified in Table S1, and were acquired between 1994 and 2018. The grounding line location (solid black line[1]), the inland limit of the drainage basin (solid grey line) and the location of transects on 14 major flow units (dashed black lines) are also shown. Profiles 1 to 9 are located on unnamed glaciers, however, 10 to 14 correspond to DeVicq, Berry, Venzke, Hull and Land Glaciers respectively. Measurements are superimposed on the BEDMAP2 bedrock topography[2].*

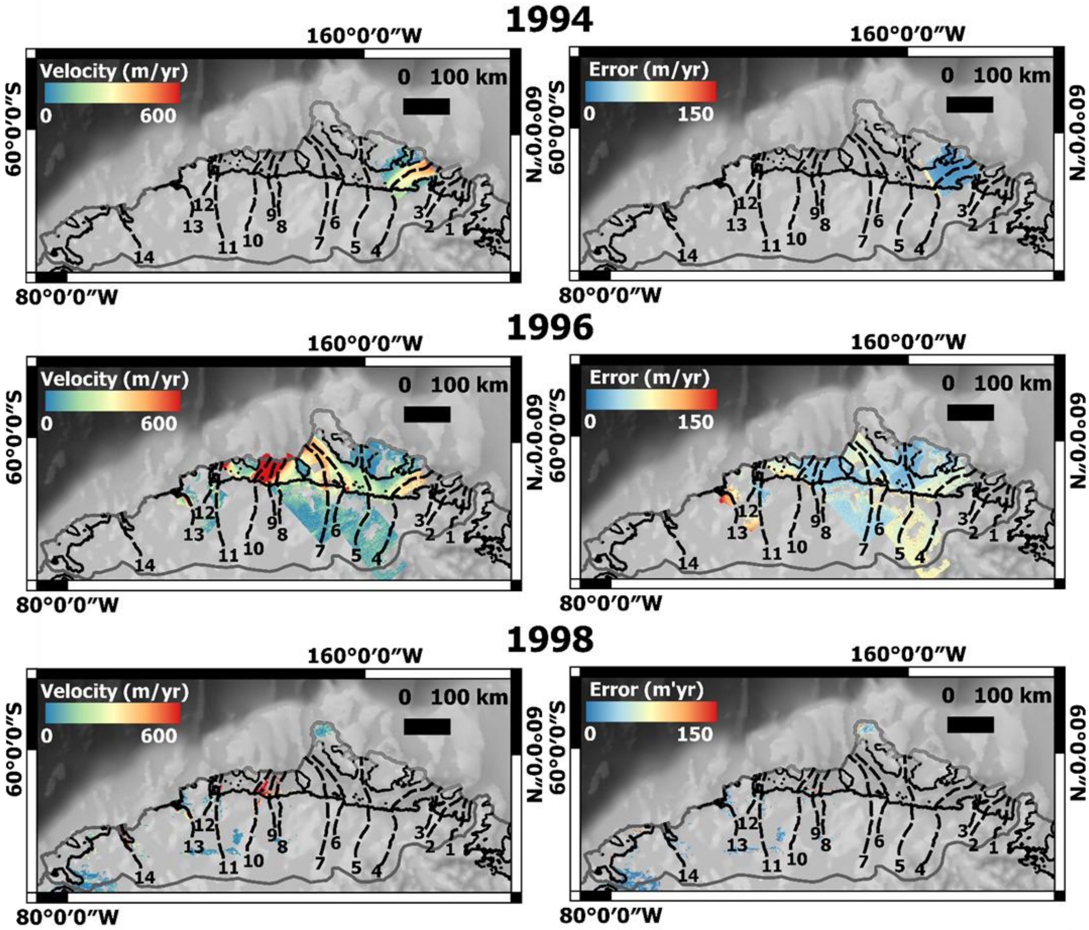

Supplementary Fig. 2b. *The observed ice speed of the Getz drainage basin (left) and the associated error estimate (right), for 2005 to 2009 annual maps. The satellite observations included in each annual map are specified in Table S1, and were acquired between 1994 and 2018. The grounding line location (solid black line[1]), the inland limit of the drainage basin (solid grey line) and the location of transects on 14 major flow units (dashed black lines) are also shown. Profiles 1 to 9 are located on unnamed glaciers, however, 10 to 14 correspond to DeVicq, Berry, Venzke, Hull and Land Glaciers respectively. Measurements are superimposed on the BEDMAP2 bedrock topography[2].*

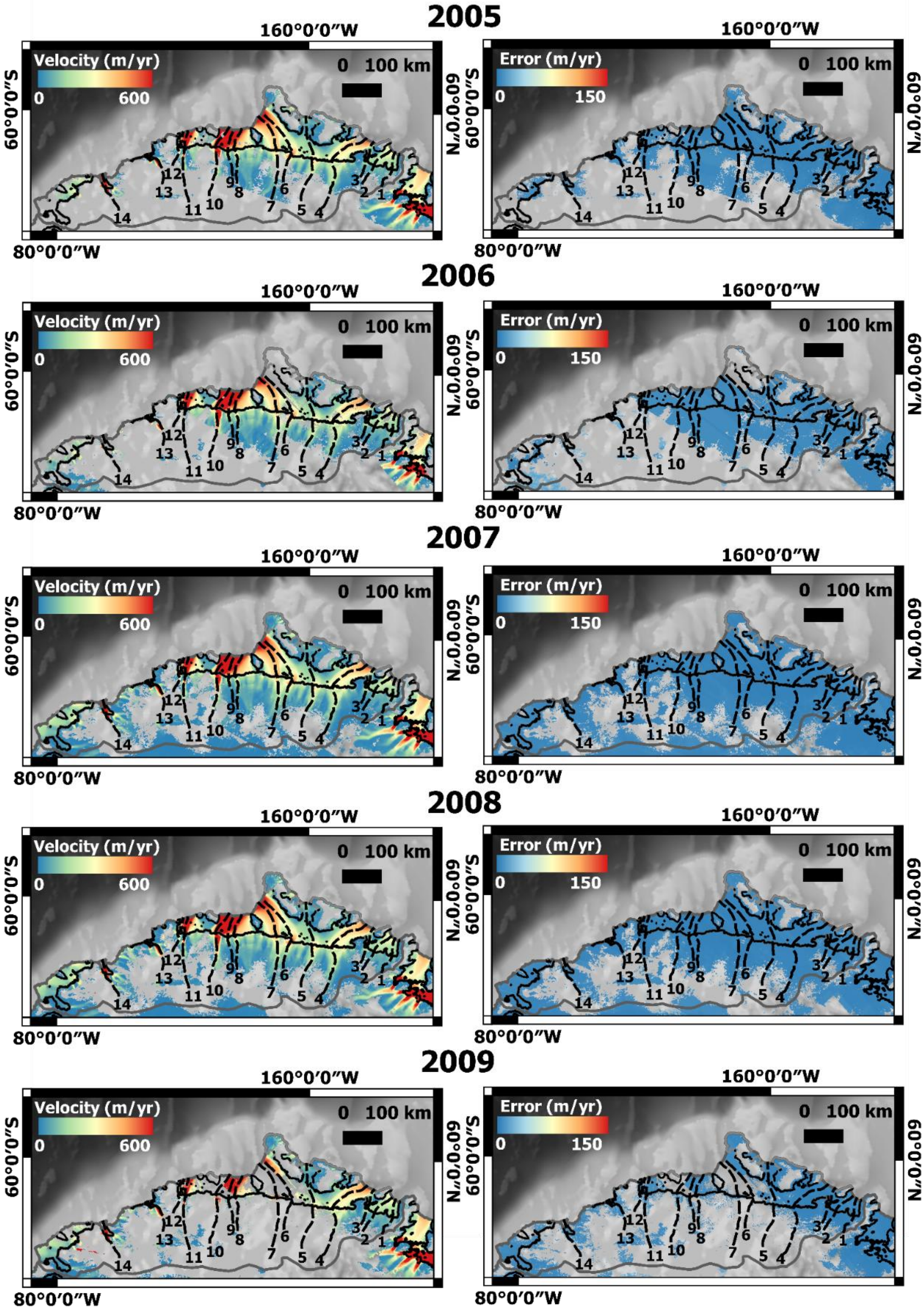

Supplementary Fig. 2c. *The observed ice speed of the Getz drainage basin (left) and the associated error estimate (right), for 2010 to 2014 annual maps. The satellite observations included in each annual map are specified in Table S1, and were acquired between 1994 and 2018. The grounding line location (solid black line*[1]*), the inland limit of the drainage basin (solid grey line) and the location of transects on 14 major flow units (dashed black lines) are also shown. Profiles 1 to 9 are located on unnamed glaciers, however, 10 to 14 correspond to DeVicq, Berry, Venzke, Hull and Land Glaciers respectively. Measurements are superimposed on the BEDMAP2 bedrock topography*[2]*.*

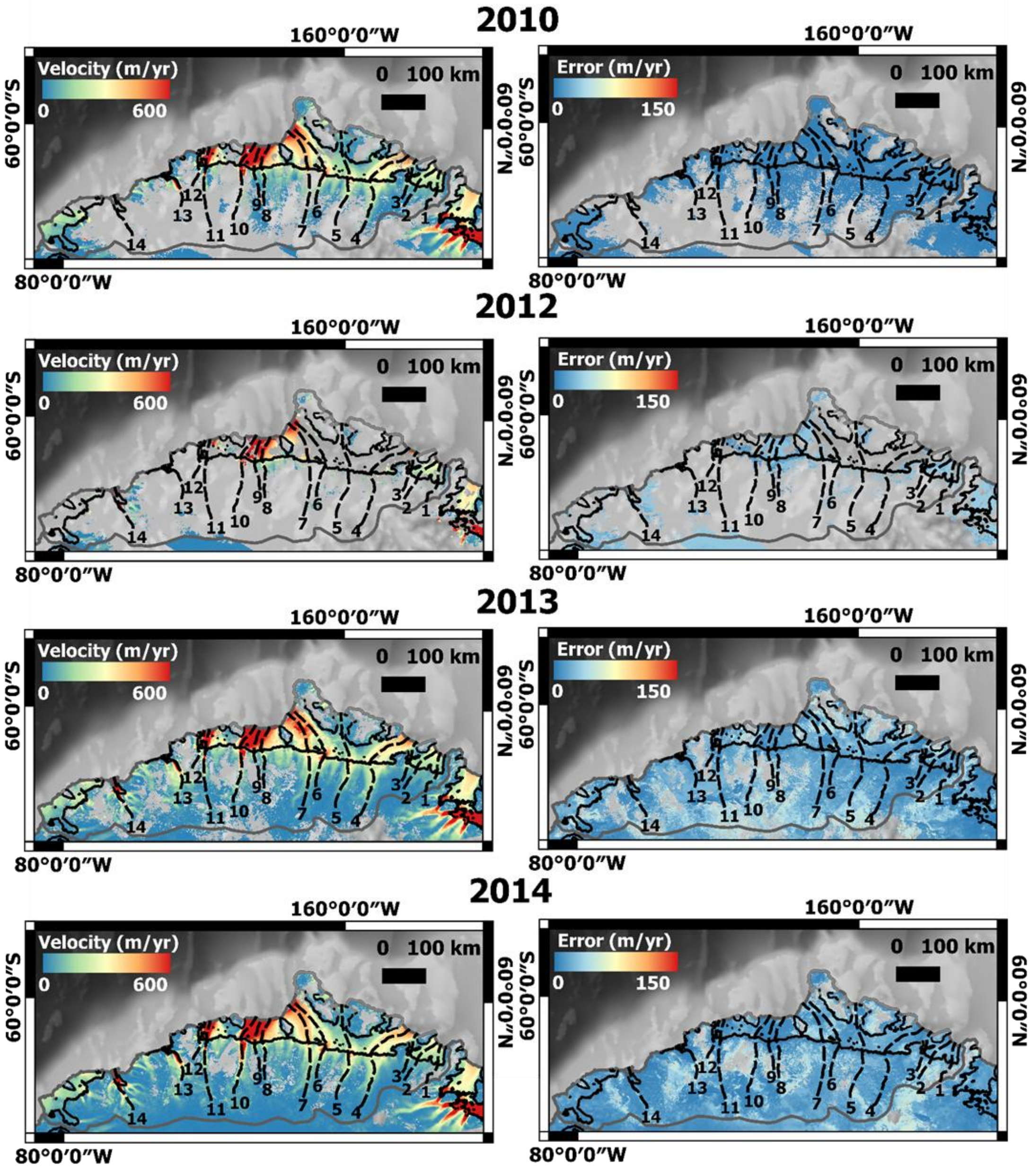

Supplementary Fig. 2d. *The observed ice speed of the Getz drainage basin (left) and the associated error estimate (right), for 2015 to 2018 annual maps. The satellite observations included in each annual map are specified in Table S1, and were acquired between 1994 and 2018. The grounding line location (solid black line[1]), the inland limit of the drainage basin (solid grey line) and the location of transects on 14 major flow units (dashed black lines) are also shown. Profiles 1 to 9 are located on unnamed glaciers, however, 10 to 14 correspond to DeVicq, Berry, Venzke, Hull and Land Glaciers respectively. Measurements are superimposed on the BEDMAP2 bedrock topography[2].*

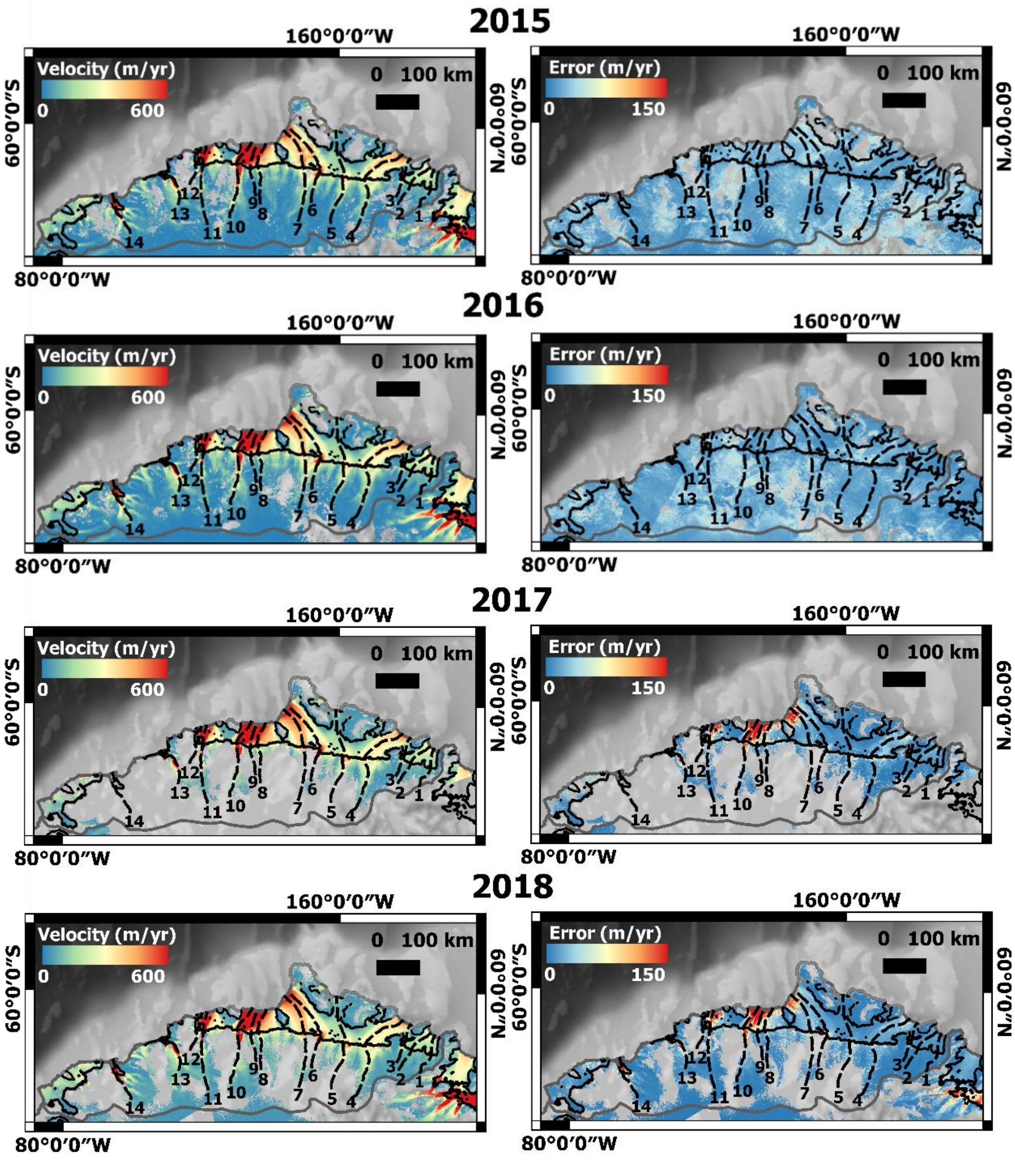

Supplementary Fig. 3. *Change in ice speed for each flow unit across the Getz Ice Basin. The observations (black circles) and the optimized model velocities (blue triangles) are shown throughout the study period. The linear fit to both datasets is also show, with good agreement between the two.*

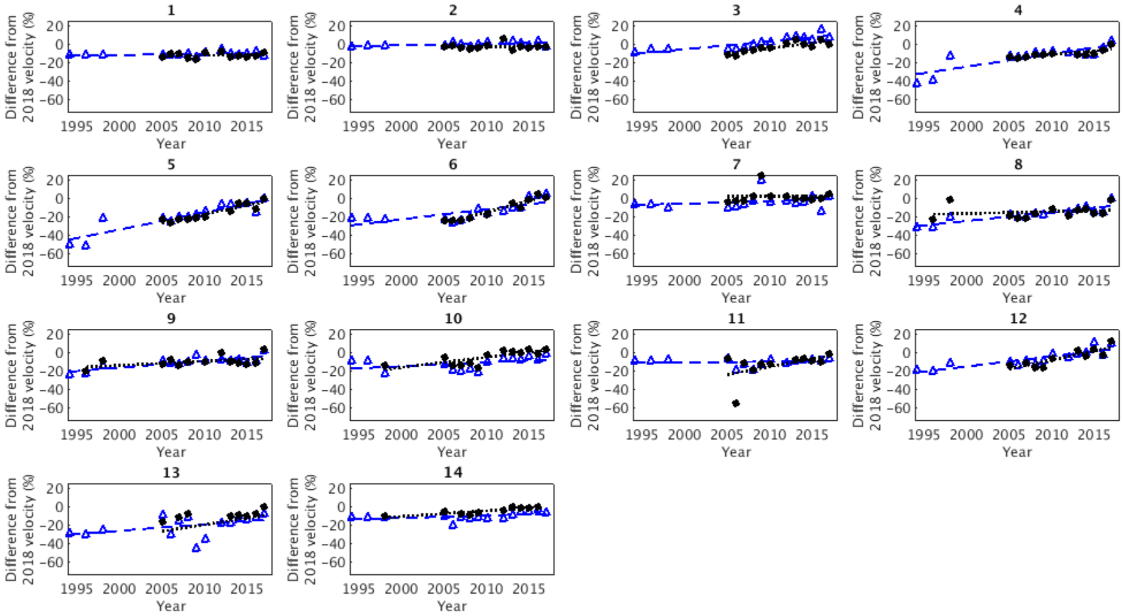

Supplementary Fig. 4a. *Profiles of the annual optimised model ice speed from 1994 (dark blue) to 2018 (light blue), extracted along flow-line transects located on the central trunk of flow unit 1 to 8 in the Getz study region. The ice surface elevation (grey line) and bed elevation (brown line), in Polar stereographic projection referenced to the WGS84 ellipsoid, extracted along the same profile from BEDMAP2[2] is also shown. The x axis is shown as distance from the grounding line[1], with positive values indicating the inland section of the profile on the ice sheet and negative values indicating seaward locations.*

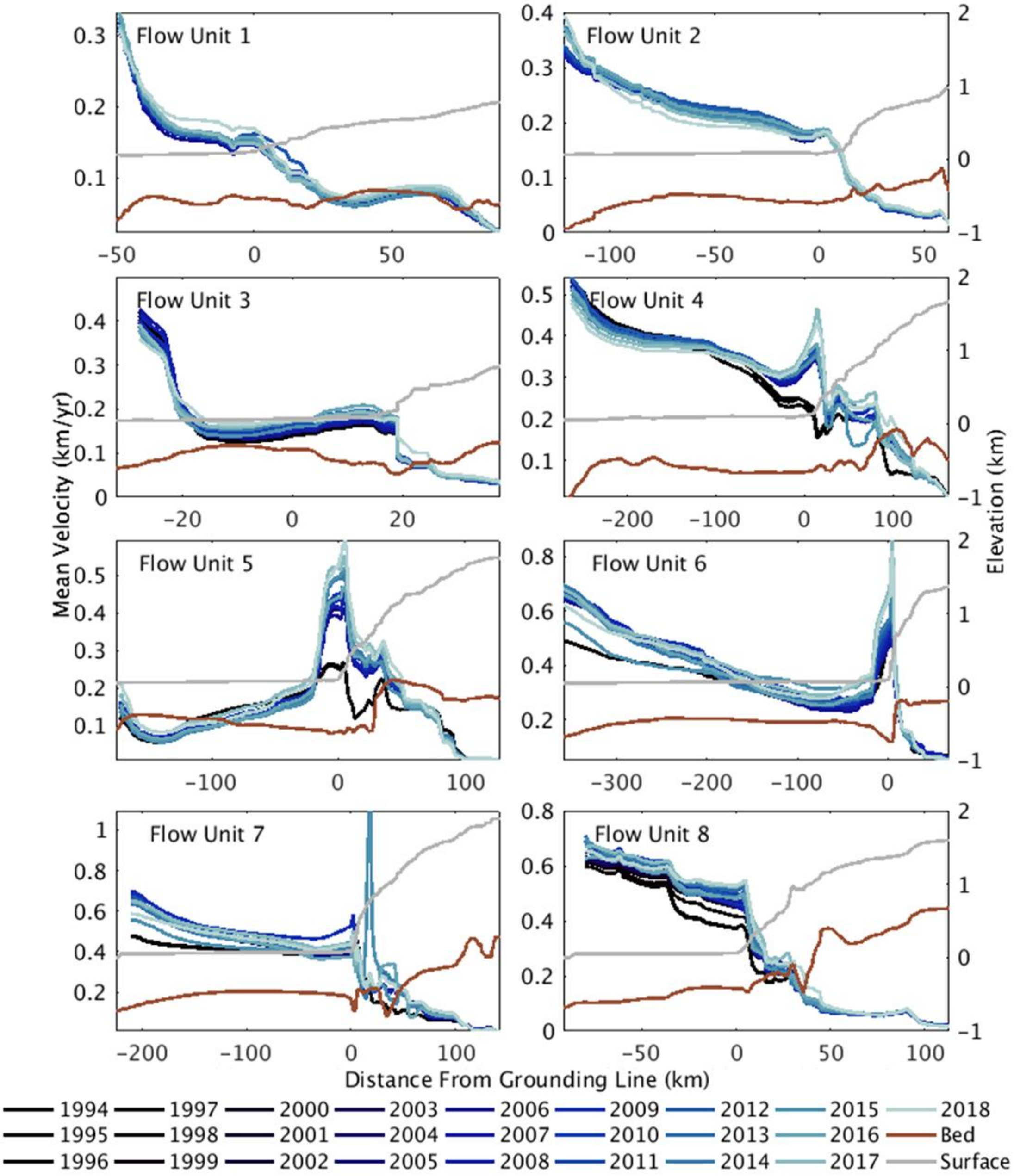

Supplementary Fig. 4b. *Profiles of the annual optimised model ice speed from 1994 (dark blue) to 2018 (light blue), extracted along flow-line transects located on the central trunk of flow unit 9 to 14 in the Getz study region. The ice surface elevation (grey line) and bed elevation (brown line), in Polar stereographic projection referenced to the WGS84 ellipsoid, extracted along the same profile from BEDMAP2[2] is also shown. The x axis is shown as distance from the grounding line[1], with positive values indicating the inland section of the profile on the ice sheet and negative values indicating seaward locations.*

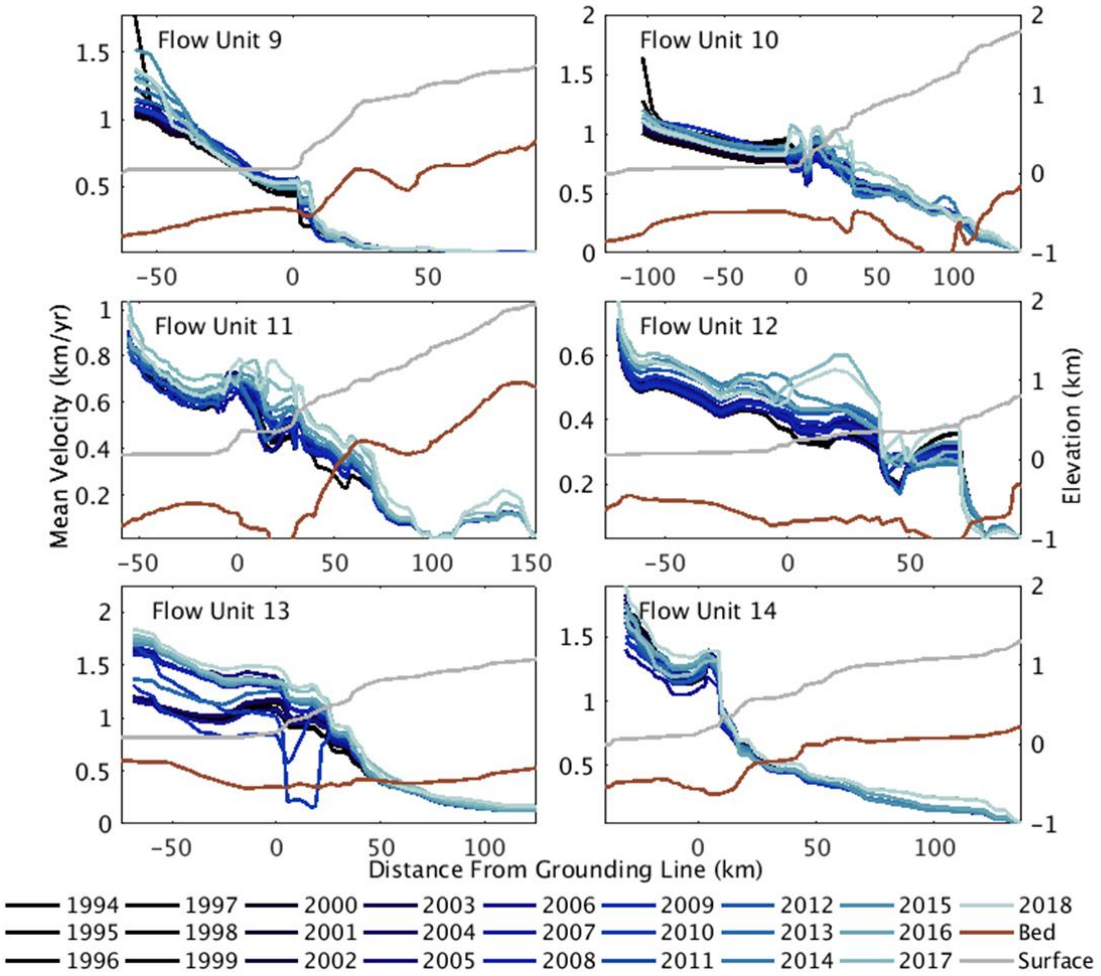

Supplementary Fig. 5. *a) Mean difference between the observed and optimised model ice speed, for every pixel in the Getz study region where corresponding measurements exist, during the 25-year study period. The mean difference across the region for each year is indicated in brackets with an average difference for all years of 11 m/yr. b) Map of the change in ice speed from the optimised model, from 1994 to 2018, calculated using the same 16 years that observations were available to enable a direct comparison. The grounding line location (solid black line[1]), inland limit of the drainage basin (solid grey line) and the location of 14 flow line profiles (dashed black lines) are also shown. Measurements are superimposed on BEDMAP2 bedrock topography[2]. c) Change in basal drag (τ), and d) relative change in the stiffening parameter (ϕ) in the Getz region from 1994 to 2018, from the optimised model.*

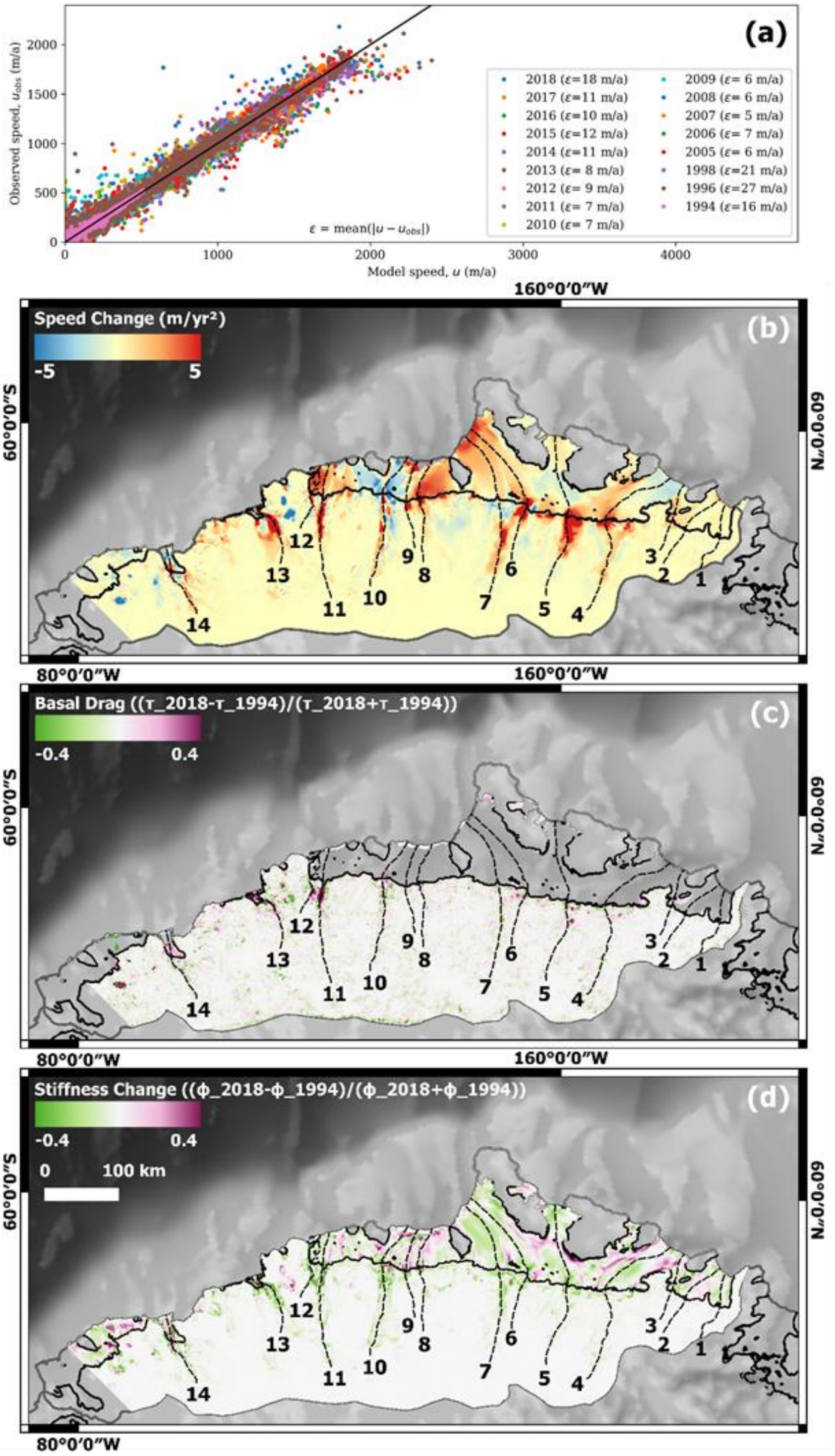

Supplementary Fig. 6a. *The optimised model ice speed in the Getz drainage basin, from 1994 to 2003. The grounding line location (solid black line[1]), the inland limit of the drainage basin (solid grey line) and the location of 14 flow unit profiles (dashed black lines) are also shown.*

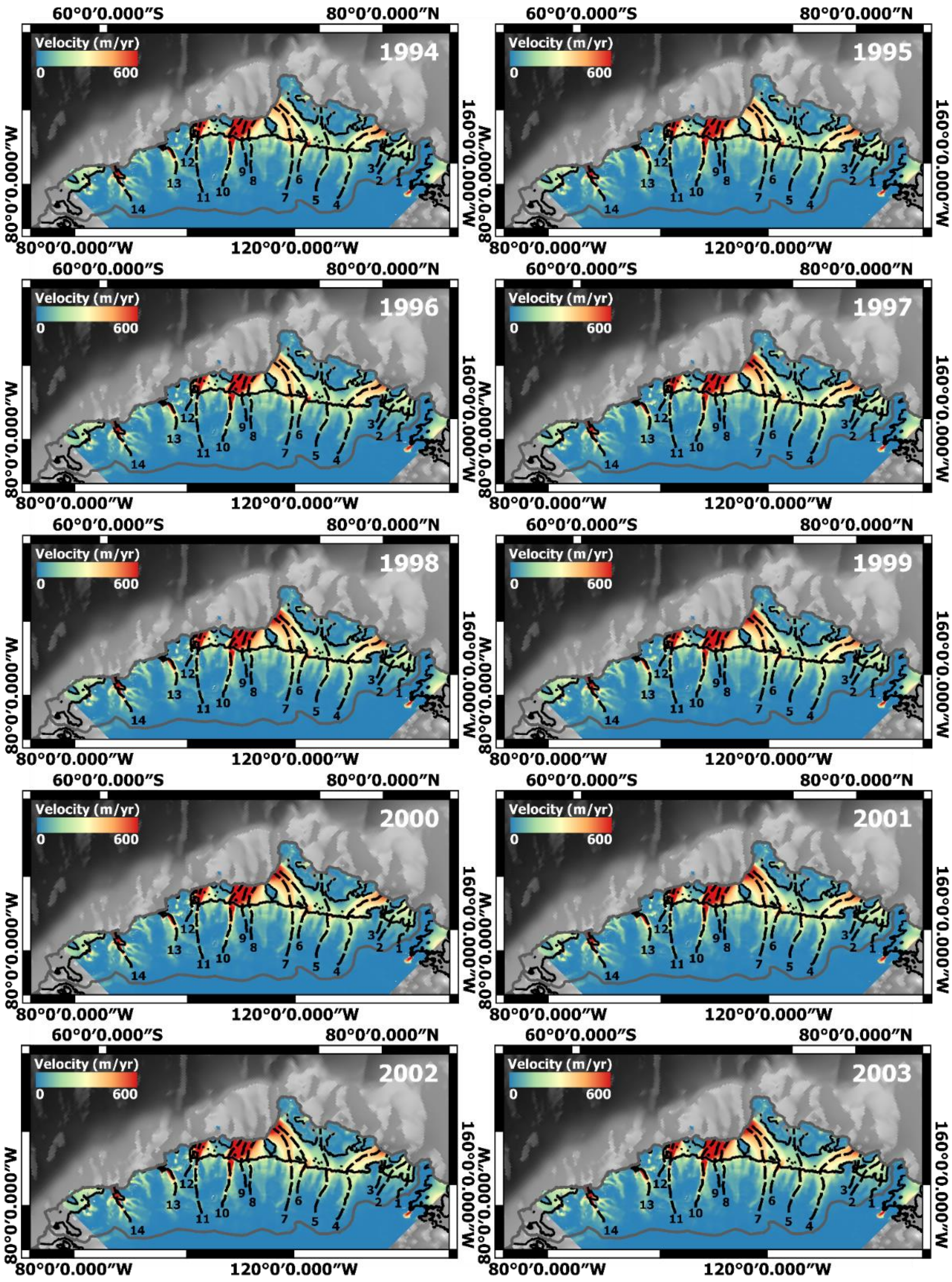

Supplementary Fig. 6b. *The optimised model ice speed in the Getz drainage basin, from 2004 to 2013. The grounding line location (solid black line[1]), the inland limit of the drainage basin (solid grey line) and the location of 14 flow unit profiles (dashed black lines) are also shown.*

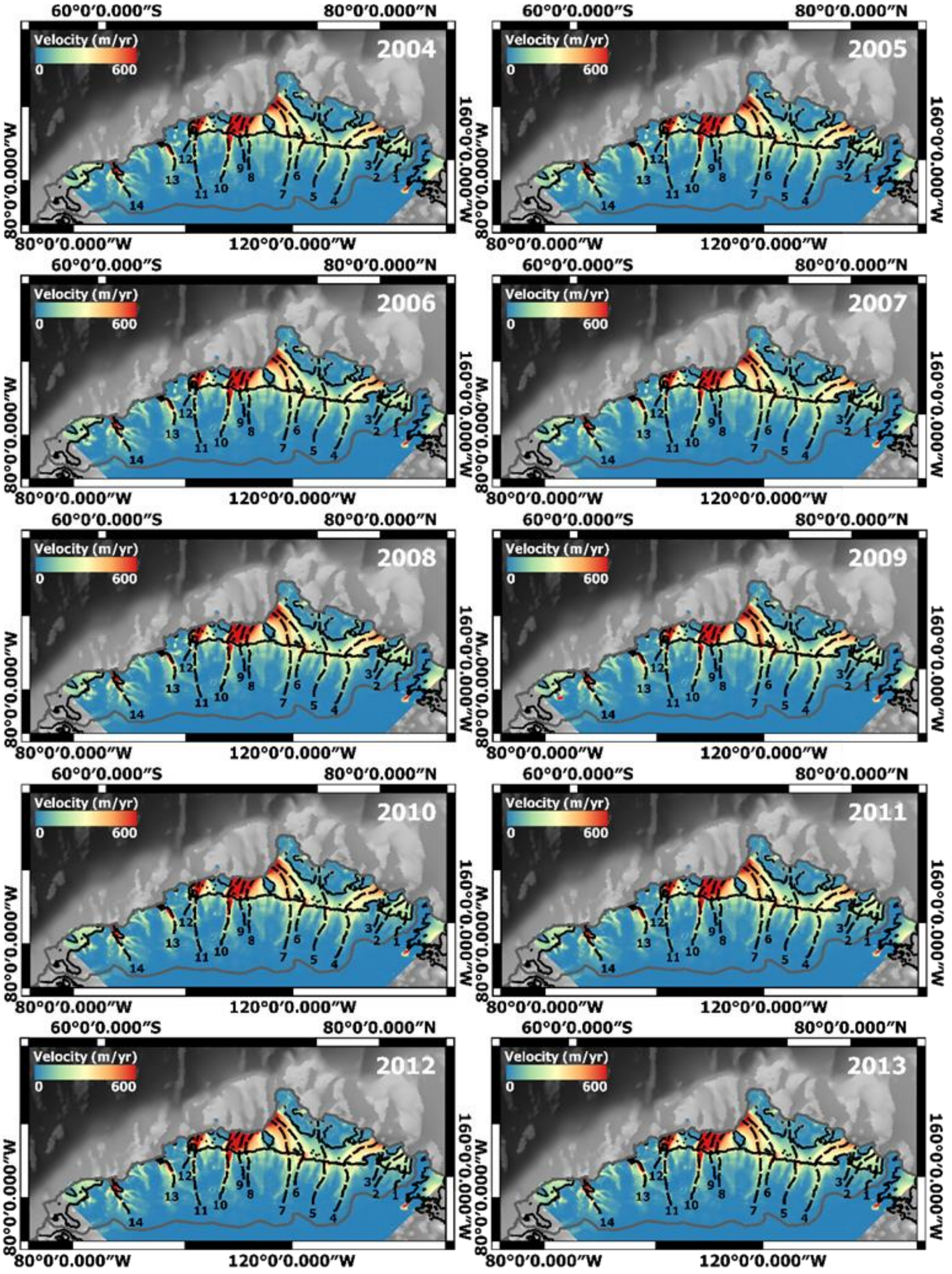

Supplementary Fig. 6c. *The optimised model ice speed in the Getz drainage basin, from 2014 to 2018. The grounding line location (solid black line[1]), the inland limit of the drainage basin (solid grey line) and the location of 14 flow unit profiles (dashed black lines) are also shown.*

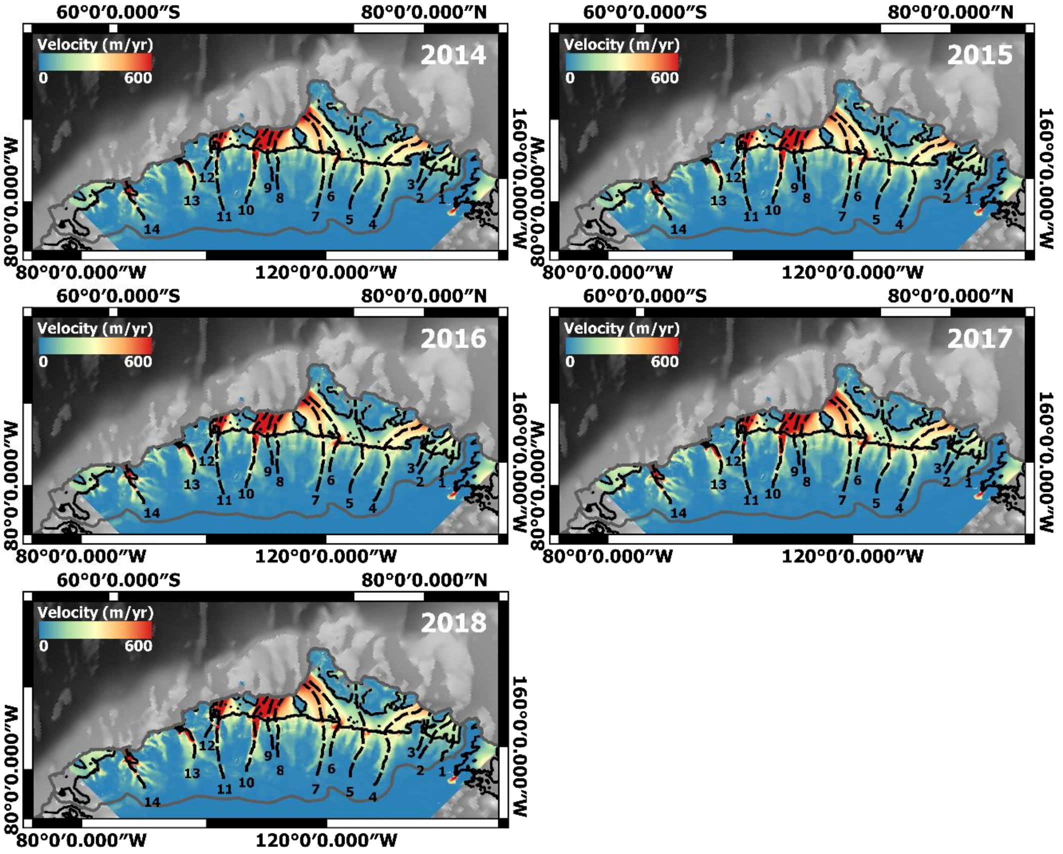

Supplementary Fig. 7. *The annual calving front location of the Getz Ice Shelf from 2009 (yellow) to 2018 (purple)*[4]*. The grounding line location (solid black line*[1]*), the inland limit of the drainage basin (solid grey line), the location of 12 flow unit profiles (dashed black lines) are also annotated, along with the location of ice rises and islands (brown area). Hull and Land Glaciers (flow units 13 and 14) do not flow into the Getz Ice Shelf, therefore they are not shown on this map.*

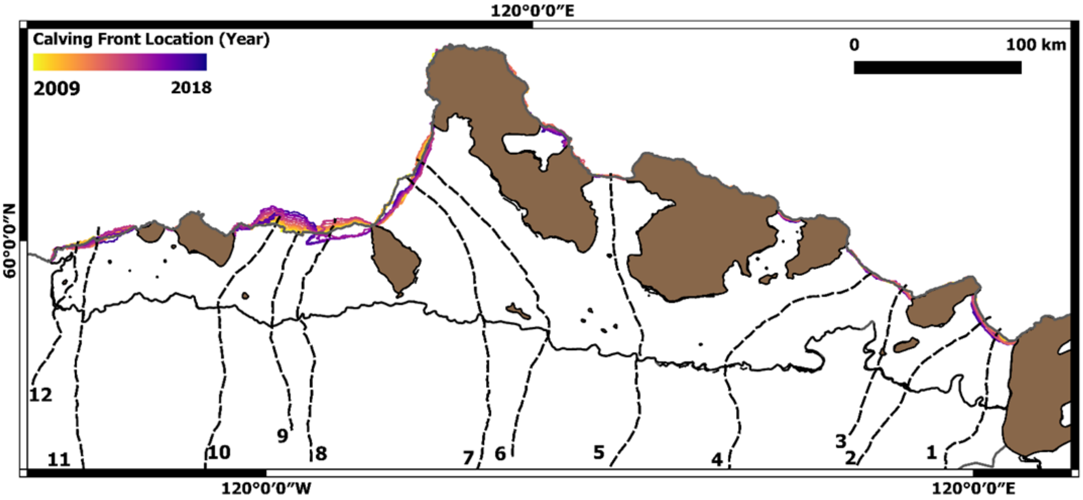

***Supplementary Fig. 8a**. Profiles of the annual observed ice speed for flow units 1 to 8 from 1994 (dark blue) to 2018 (light blue), extracted along flow-line transects located on the central trunk of 14 glaciers in the Getz study region. The ice surface elevation (grey line) and bed elevation (brown line), in Polar stereographic projection referenced to the WGS84 ellipsoid, extracted along the same profile from BEDMAP2[2] is also shown. The x axis is shown as distance from the grounding line[1], with positive values indicating the inland section of the profile on the ice sheet and negative values indicating seaward locations.*

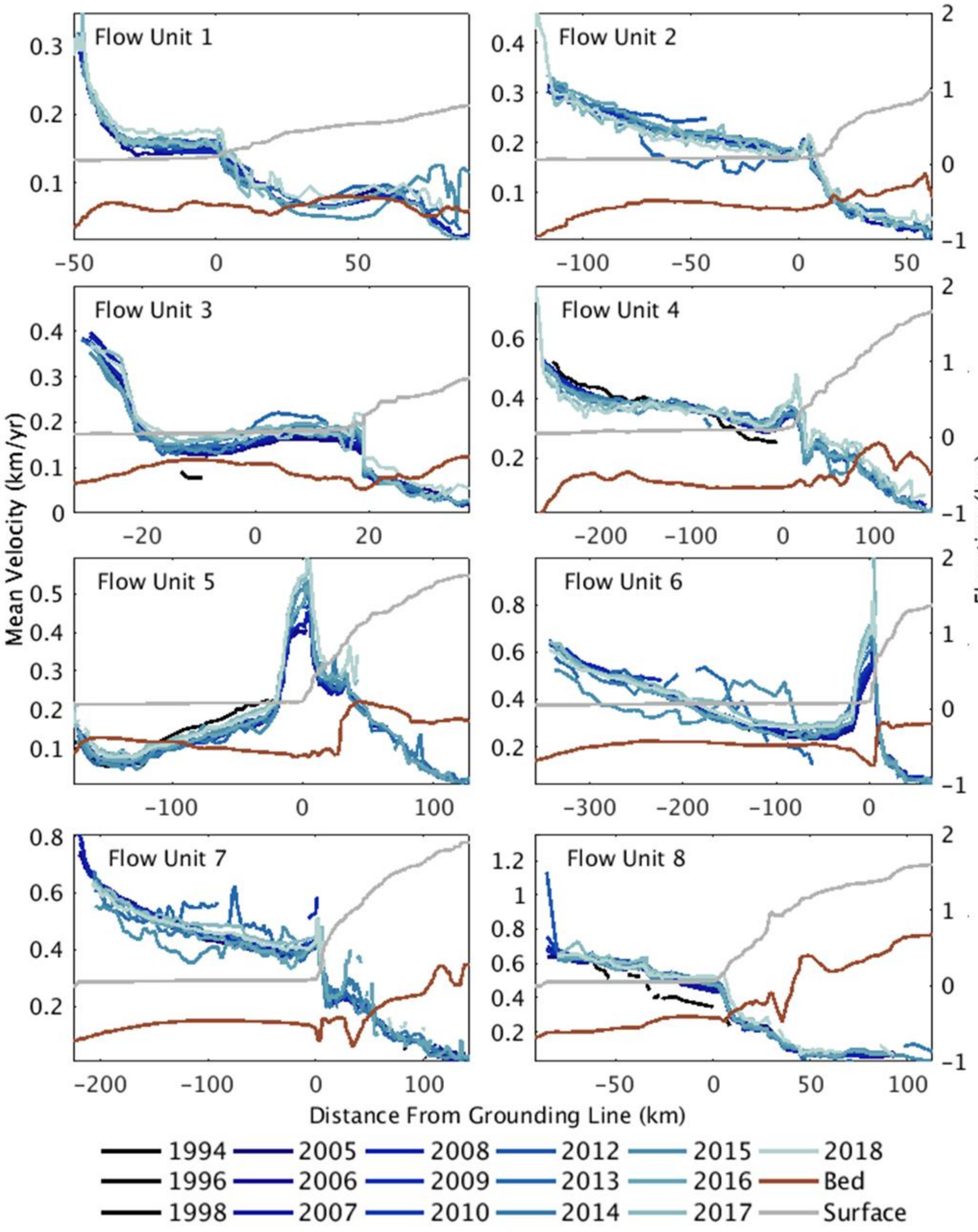

Supplementary Fig. 8b*. Profiles of the annual observed ice speed for flow units 9 to 14 from 1994 (dark blue) to 2018 (light blue), extracted along flow-line transects located on the central trunk of 14 glaciers in the Getz study region. The ice surface elevation (grey line) and bed elevation (brown line), in Polar stereographic projection referenced to the WGS84 ellipsoid, extracted along the same profile from BEDMAP2[2] is also shown. The x axis is shown as distance from the grounding line[1], with positive values indicating the inland section of the profile on the ice sheet and negative values indicating seaward locations.*

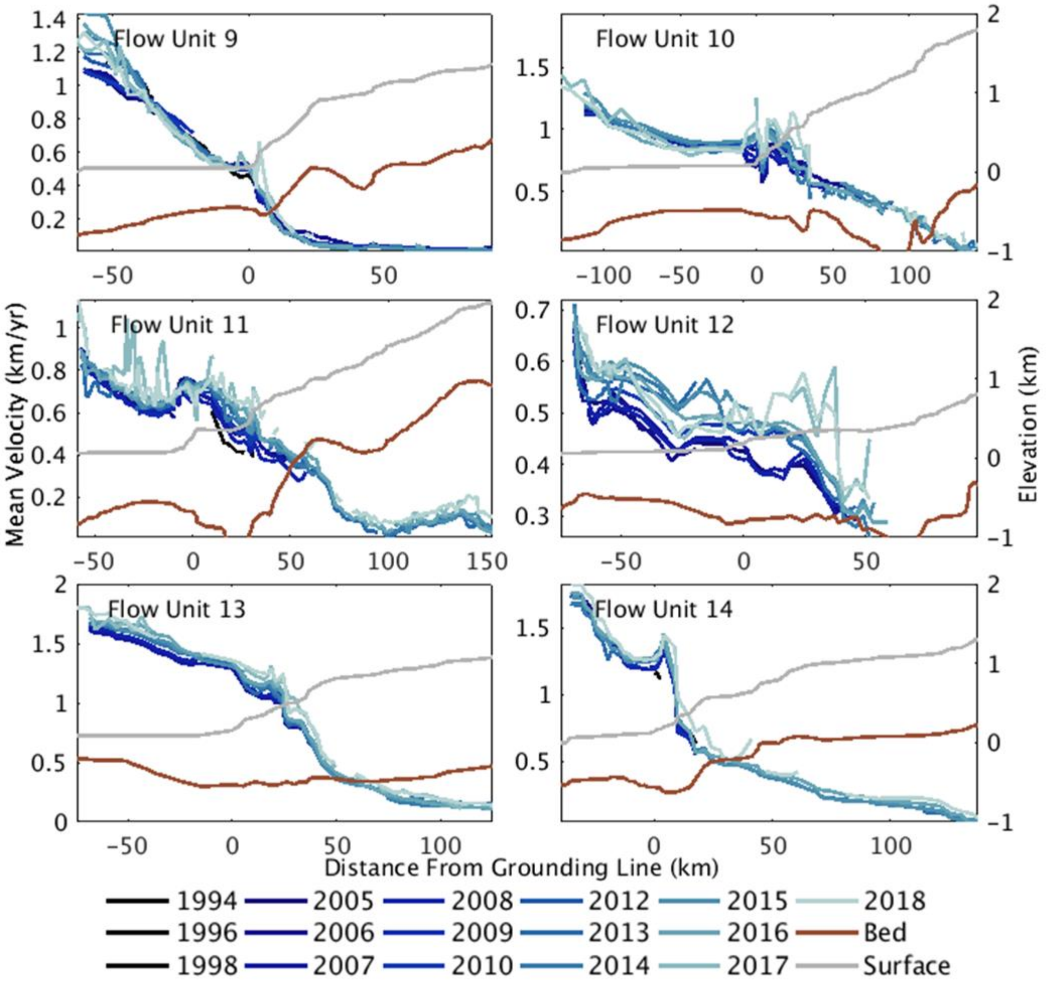

Supplementary References

1. Rignot, E., Mouginot, J., B. S. MEaSUREs Antarctic Grounding Line from Differential Satellite Radar Interferometry, Version 2. *Boulder, Color. USA. NASA Natl. Snow Ice Data Cent. Distrib. Act. Arch. Center. doi/10.5067/IKBWW4RYHF1Q* (2016).

2. Fretwell, P. *et al.* Bedmap2: Improved ice bed, surface and thickness datasets for Antarctica. *Cryosphere* **7,** 375–393 (2013).

3. Shepherd, A. *et al.* Trends in Antarctic Ice Sheet Elevation and Mass. *Geophys. Res. Lett.* **46,** 8174–8183 (2019).

4. Andreasen, J. Hogg, A.E. & Selley, H.L. Change in area of Antarctic Ice Shelves from 2009 to 2019. Manuscript in preparation
